# Potential of Bacteria from Alternative Fermented Foods as Starter Cultures for the Production of Wheat Sourdoughs

**DOI:** 10.3390/microorganisms8101534

**Published:** 2020-10-06

**Authors:** Andrea Comasio, Simon Van Kerrebroeck, Henning Harth, Fabienne Verté, Luc De Vuyst

**Affiliations:** 1 Research Group of Industrial Microbiology and Food Biotechnology (IMDO), Faculty of Sciences and Bioengineering Sciences, Vrije Universiteit Brussel (VUB), Pleinlaan 2, B-1050 Brussels, Belgium; andrea.comasio@vub.be (A.C.); Simon.Van.Kerrebroeck@vub.be (S.V.K.); Henning.Harth@vub.be (H.H.); 2 Puratos NV, Industrialaan 25, 1702 Groot-Bijgaarden, Belgium; FVerte@puratos.com

**Keywords:** sourdough, *Lactobacillaceae*, acetic acid bacteria, *Gluconobacter oxydans*, *Acetobacter pasteurianus*, *Staphylococcus carnosus*, bread

## Abstract

Microbial strains for starter culture-initiated sourdough productions are commonly isolated from a fermenting flour–water mixture. Yet, starter culture strains isolated from matrices other than sourdoughs could provide the dough with interesting metabolic properties and hence change the organoleptic properties of the concomitant breads. Furthermore, the selection of sourdough starter cultures does not need to be limited to lactic acid bacteria (LAB), as other food-grade microorganisms are sometimes found in sourdoughs. Therefore, different strains belonging to LAB, acetic acid bacteria (AAB), and coagulase-negative staphylococci (CNS) that originated from different fermented food matrices (fermenting cocoa pulp-bean mass, fermented sausage, and water kefir), were examined as to their prevalence in a wheat sourdough ecosystem during 72-h fermentations. *Limosilactobacillus fermentum* IMDO 222 (fermented cocoa pulp-bean mass isolate) and *Latilactobacillus sakei* CTC 494 (fermented sausage isolate) seemed to be promising candidates as sourdough starter culture strains, as were the AAB strains *Acetobacter pasteurianus* IMDO 386B and *Gluconobacter oxydans* IMDO A845 (both isolated from fermented cocoa pulp-bean mass), due to their competitiveness in the wheat flour-water mixtures. Wheat breads made with *G. oxydans* IMDO A845 sourdoughs were significantly darker than reference wheat breads.

## 1. Introduction

Sourdough is a specific and stressful microbial ecosystem, inhabited by mainly yeasts and lactic acid bacteria (LAB), which is obtained through fermentation of a flour–water mixture and used for the production of baked goods [1,2,3,4,5,6,7,8,9,10,11]. During spontaneous fermentation, these microorganisms come from the flour, other dough ingredients, and/or the environment [12,13,14]. Through a backslopping process, a stable microbiota that is adapted to the process conditions applied is selected [1,2,15,16,17,18,19]. Traditionally, sourdoughs are backslopped, at least daily, at moderate temperature (room temperature). These firm sourdoughs are called Type I or Type 1 sourdoughs [3,20]. Additionally, starter cultures can be applied, enabling direct (long) fermentations without backslopping [2,3,21,22]. This is often the case for industrially produced sourdoughs, fermented at a temperature of at least 30 °C and for one to three days [3,23]. These liquid to semi-liquid sourdoughs are classified as Type II or Type 2 sourdoughs [2,3,20]. Dried preparations of starter culture-initiated (Type II/2) sourdoughs are referred to as Type III sourdoughs [2,3,20]. Starter culture-initiated sourdoughs that are further backslopped are called Type 3 sourdoughs [2,3].

Sourdough-specific heterofermentative lactobacilli are usually preferred to produce starter culture-initiated sourdoughs as bacterial strains originally isolated from a sourdough matrix are supposed to possess specific adaptations to this ecosystem, thus enhancing their competitiveness [2,3,24]. Examples of such adaptations are the heterofermentative degradation of maltose and the use of fructose as an alternative external electron acceptor, as well as the expression of the arginine deiminase pathway. As such, sourdough-specific strains of species belonging to the *Lactobacillaceae* family have been isolated and tested as candidate starter cultures for sourdough production processes, in particular with respect to the use of non-conventional flours [25,26,27,28,29,30,31,32,33,34,35]. Similarly, alternative yeast species can be exploited to produce innovative sourdoughs [1,26,33].

However, the phenotypic traits mentioned above are not necessarily limited to sourdough-isolated strains [34]. Moreover, food-grade microorganisms originating from food matrices other than sourdoughs and/or different from LAB could provide interesting metabolic properties to sourdoughs and hence change the organoleptic properties of the concomitant breads. Therefore, microorganisms originating from other fermented food matrices, such as fermenting cocoa pulp-bean mass, fermented sausage, and water kefir, whether or not belonging to species or genera frequently isolated from sourdoughs, are worth investigating regarding their use as candidate starter cultures, as strains of many of these species have been studied thoroughly [35,36,37,38,39,40,41,42,43,44]. Specialized metabolic traits needed for their prevalence in these non-sourdough matrices could confer additional metabolic flexibilities to these strains in the sourdough ecosystem, provided they can grow and be metabolically active in the latter, and hence further contribute to the final quality of the sourdoughs and breads produced therefrom. As such, starter culture strains have been tested that were isolated from, for instance, the cocoa fermentation process [28] or kimchi [45].

The alternative fermented food ecosystems mentioned above are of particular interest. Strains of *Limosilactobacillus fermentum* prevail during the LAB phase of the three-step cocoa fermentation process, consuming carbohydrates and citrate and producing lactic acid, acetic acid, mannitol, and buttery-flavor compounds [35,46,47,48]. Meat fermentations for fermented sausage production are mainly carried out by *Latilactobacillus sakei* acidifying the meat batter and, in some cases, producing bacteriocins against spoiling and pathogenic bacteria [42,49]. Non-conventional LAB species, such as *Lentilactobacillus hilgardii* and *Liquorilactobacillus nagelii*, prevail during water kefir fermentation processes, during which growth of water kefir grains made of exopolysaccharides occurs and lactic acid, acetic acid, ethanol, and mannitol are produced [38,39,50,51].

Furthermore, bacterial groups other than LAB, in particular acetic acid bacteria (AAB), can be considered [3]. Indeed, AAB are present in certain sourdoughs, indicating their potential for acetic acid production [32,52,53,54]. Their application as starter cultures has been examined, mainly, regarding in situ production of exopolysaccharides [55,56,57]. Only few studies report on their flavor-forming potential [57,58]. Besides LAB and AAB, other bacterial groups have not been examined up to now, as they do not prevail during sourdough productions. For instance, coagulase-negative staphylococci (CNS), which play a role in meat fermentation processes regarding aroma and color development [49,59] and can be present in the original flour [3], have not been considered. With respect to their metabolism, their unique combinations of glucose breakdown and amino acid conversions could contribute to the production of desirable flavors [60,61,62].

The aim of this study was to examine the potential of dedicated well-studied bacterial strains, previously isolated from different food fermentation matrices other than sourdoughs, as non-conventional starter cultures for Type II/2 sourdough production to assess their growth in a sourdough matrix and their impact on the organoleptic properties of the concomitant breads produced. 

## 2. Materials and Methods

### 2.1. Flour

All sourdough production was performed with one batch of wheat flour originating from a Belgian flour mill (Ceres, Vilvoorde, Belgium). Based on the information provided by the commercial source, it contained, on average (*m/m*), 15.0% moisture, 71.0% carbohydrates, 12.0% proteins, 1.5% dietary fiber, 1.0% fat, and 0.54–0.60% ash. The Hagberg falling number was above 250 s.

### 2.2. Starter Culture Strains, Inoculum Build-up, and Enumeration

An overview of the non-conventional bacterial strains used throughout this study is given in Table 1. The strains selected have been studied thoroughly in the past, as indicated by the concomitant references (Table 1).

Inocula of these starter culture strains were produced as described previously [25]. Briefly, the strains were propagated at 30 °C in 10 mL of the appropriate media for 24 h, namely modified de Man–Rogosa–Sharpe (mMRS-5) medium for LAB [64], mannitol-yeast extract-peptone (MYP) medium for AAB [40], and brain heart infusion (BHI) medium for CNS (Oxoid, Basingstoke, Hampshire, UK) [43]. A second optimized propagation step (transfer volume of 1%, *v/v*) was performed at 30 °C in an appropriate volume to achieve an initial density of at least 10^6^–10^7^colony forming units (CFU) per mL of wheat flour–water mixture.

Solid media were prepared by adding 1.5% (*m/v*) agar (Oxoid) to the appropriate liquid media and were used for the enumeration of LAB, AAB, CNS, and yeasts. For LAB and CNS, mMRS-5 agar and mannitol salt agar (MSA) were used, respectively. Both media were supplemented with 100 ppm of cycloheximide (Sigma-Aldrich, Saint Louis, MO, USA) and 5 ppm of amphotericin B (Sigma-Aldrich). For AAB and yeasts, modified deoxycholate-mannitol-sorbitol (mDMS) agar medium supplemented with 100 ppm of cycloheximide (Sigma-Aldrich) and 5 ppm of amphotericin B (Sigma-Aldrich) [65] and yeast extract-peptone-dextrose (YPD) agar medium containing 100 ppm of chloramphenicol (Sigma-Aldrich) [66] were used, respectively. Bacterial and yeast counts were determined after 72 h of incubation at 30 °C and expressed as CFU/mL.

### 2.3. Sourdough Production

Starter culture-initiated liquid wheat sourdough productions (Type II/2) were performed at 30°C for 72 h in 15-L stainless steel laboratory fermenters (Biostat C; Sartorius, Melsungen, Germany), as described previously [25]. The respective codes of these sourdough production processes are indicated in Table 1. The productions were done in single, duplicate, or triplicate, depending on the success of the candidate starter culture strains used. Briefly, for each sourdough production, 2 kg of wheat flour was added to the fermenter, which was pre-sterilized (121 °C, 2.1 bar, 20 min) with 6 L of soft water, equaling a sourdough mass of 8 kg with a dough yield [DY; (dough mass/flour mass) × 100] of 400. The mixture was kept homogeneous by continuous agitation (300 rpm). The fermenters were flushed with 1 L/min of sterile air through their headspace to mimic the microaerophilic conditions of industrial sourdough fermentations, except for the oxygen-requiring AAB-sourdough initiated fermentation processes, during which 5 L/min of sterile air was sparged through the flour-water mixtures (except for GO3 that was aerated through the headspace; Table 1). Depending on the strain tested, different modifications of the general fermentation conditions and/or ingredients were applied, as explained in Table 1.

Samples were withdrawn from the fermenters aseptically before their inoculation with the starter culture strain under study (further referred to as time 0), after inoculation (time 0′), and after 24, 48, and 72 h of fermentation, and were further processed as described previously [15,25].

### 2.4. Sample Processing

#### 2.4.1. Determination of pH and Total Titratable Acidity

Sourdough pH and total titratable acidity (TTA) were determined making use of fresh fermentation samples, as described previously [15]. The pH was measured with an InoLab 720 pH meter (WTW, Weilheim, Germany). The TTA value was measured by titration of a suspension of 10 g of sourdough in 100 mL of ultrapure water (MilliQ Merck Millipore, Burlington, MA, USA) with 0.1 M NaOH; the TTA value was expressed as the number of mL of 0.1 M NaOH used to titrate the sample to reach a final pH of 8.5.

#### 2.4.2. Microbiological Analysis of Sourdough Fermentation Samples

Culture-dependent microbiological analysis (plating and incubation, followed by colony picking and identification, as well as plate washes) and culture-independent microbial community profiling (based on denaturing gradient gel electrophoresis (DGGE) of rRNA-targeted PCR amplicons) of the fermentation samples were performed as described previously [25]. Briefly, plating of dilutions of 10 g of fresh sample, mixed with 90 mL of saline (0.85%, *m/v*, NaCl (Merck, Darmstadt, Germany)) in a stomacher bag (Stomacher 400, Seward, Worthington, Leicestershire, UK), were performed on the agar media mentioned above, which were incubated at 30 °C for 72 h. A maximum number of 10 colonies of the appropriate microorganisms were randomly picked from appropriate dilutions on the agar media for molecular identification ((GTG)_5_-PCR (bacteria) and M13-PCR (fungi) fingerprinting analysis of genomic DNA, followed by 16S rRNA gene (bacteria) and internal transcribed spacer (ITS) region sequencing of cluster representatives). Plating was performed at every time point, except for the sourdough productions initiated with *Lenl. hilgardii* IMDO 2M0G15 (LH2), *St. carnosus* IMDO F4P1 (SC2), *G. oxydans* IMDO A845 (GO1), and *A. pasteurianus* IMDO 386B (AP), which were plated only at the beginning and after 72 h of fermentation. Microbial identification was performed only for isolates picked from the appropriate agar media after 72 h of fermentation, except for the *St. carnosus* IMDO F4P1-initiated sourdough production SC1, during which no isolates were picked, and the *G. oxydans* IMDO A845-initiated sourdough production GO1 and GO2 where no isolates could be picked. For the plate washes, cells were resuspended and collected from the appropriate agar media (corresponding with the 100 × dilutions) using 10 mL of peptone-physiological solution. These mixtures were centrifuged (4600 × *g* for 20 min at 4 °C), the supernatants were discarded, and the cell pellets were stored at −20 °C. Bacterial or fungal DNA was further extracted and analyzed using PCR-DGGE as described previously [25]. Briefly, the V3 region of the 16S rRNA gene (bacteria) or the D1 region of the 26S rRNA gene (yeasts) of the extracted DNA were amplified through PCR. The PCR amplicons were separated using a denaturing gradient of formamide (Sigma-Aldrich) and urea (National Diagnostics, Atlanta, GA, USA). The DNA bands of interest were excised from the gel and subjected to a second PCR amplification. The PCR products obtained were purified with a Wizard^®^ SV Gel and PCR Clean-Up System (Promega, Madison, WI, USA) and sequenced in a commercial facility by means of capillary sequencing technology (VIB Genetic Service Facility, Antwerp, Belgium). A BLAST analysis was performed to determine the closest known relatives of the partial sequences obtained in the non-redundant nt database of the National Centre for Biotechnological Information (http://www.ncbi.nlm.nih.gov/BLAST/). Sequence identities of ≥98% were taken into account. Finally, DNA extracted from cell pellets derived from fermentation samples of 10 g, supplemented with 90 mL of peptone-physiological solution, were subjected to PCR-DGGE too, as described above [25].

#### 2.4.3. Metabolite Target Analysis to Map Substrate Consumption and Metabolite Production

Supernatants of 50 g of sample were subjected to metabolite target analysis. The determination of carbohydrate (fructose, glucose, maltose, and sucrose) and sugar alcohol (erythritol, glycerol, mannitol, and sorbitol) concentrations in cell-free culture supernatants of the fermentation samples was performed through high-performance anion exchange chromatography with pulsed amperometric detection (HPAEC-PAD) making use of an ICS 3000 chromatograph equipped with an AS-autosampler and a CarboPac PA10 column (Dionex, Sunnyvale, CA, USA); that of acetic acid, acetoin, diacetyl, and ethanol through gas chromatography with flame ionization detection (GC-FID) making use of a Focus gas chromatograph (Interscience, Breda, The Netherlands), equipped with a Stabilwax-DA column (Restek, Bellefonte, PA, USA), a FID-80 detector (Interscience), and an AS 3000 autosampler (Interscience); that of lactic acid (L and D) through high-performance liquid chromatography with ultraviolet detection (HPLC-UV) making use of a Waters chromatograph (Waters, Milford, MA, USA), equipped with a 600S controller, a 717Plus autosampler, a 486 UV detector (set at a wavelength of 253 nm), and a Shodex Orpak CRX-853 column (Showa Denko, Tokyo, Japan), as described previously [25].

The determination of volatile organic compounds was performed through headspace/solid-phase microextraction GC coupled to mass spectrometry (HS/SPME-GC-MS), making use of an Agilent 6890 gas chromatograph coupled to an Agilent 5973 N mass spectrometer (Agilent Technologies, Santa Clara, CA, USA), an MPS2 Gerstel autosampler (Gerstel, Mülheim-an-der-Ruhr, Germany), a 75-μm SPME divinylbenzene/carboxen/polydimethylsiloxane (DVS/CAR/PDMS) fiber (Agilent Technologies), and a DB-WAXetr capillary column (Agilent Technologies), as described previously [15]. Principal component analysis (PCA) was carried out on the normalized peak areas of the volatile organic compounds detected in the sourdoughs using the SPSS 20.0 software package for Windows (IBM, Armonk, NY, USA). Therefore, Varimax rotation with Kaiser normalization was performed.

For the AAB-initiated sourdough productions, the concentrations of gluconic acid, 2-ketogluconic acid, and 5-ketogluconic acid were determined using HPAEC with conductivity under ion suppression (HPAEC-CIS), as described previously, with some modifications [35]. Therefore, an ICS 3000 chromatograph equipped with an AS-19 column (Dionex, Sunnyvale, CA, USA) was used. The column temperature was set at 30 °C. The flow rate of the mobile phase was 1.0 mL/min and consisted of ultrapure water (MilliQ; eluent A) and 100 mM KOH (eluent B). The following gradient was applied: 0 min, 99% A and 1% B; 17 min, 99% A and 1% B; 25 min, 96% A and 4% B; 35 min, 80% A and 20% B; 42 min, 75% A and 25% B; 45 min, 70% A and 30% B; 47 min, 70% A and 30% B; 48 min, 0% A and 100% B; 54 min, 0% A and 100% B; 55 min, 99% A and 1% B; 65 min, 0% A and 100% B. A standard addition protocol was applied. Therefore, four standard solutions with the following compositions were prepared (g/L): ultrapure water (MilliQ; solution A); 0.50 gluconic acid, 0.25 2-ketogluconic acid, and 0.25 5-ketogluconic acid (solution B); 1.00 gluconic acid, 0.50 2-ketogluconic acid, 0.50 5-ketogluconic acid (solution C); 1.50 gluconic acid, 0.75 2-ketogluconic acid, and 0.75 5-ketogluconic acid (solution D). Subsequently, 300 μL of the supernatants was mixed with 600 μL of acetonitrile (Sigma-Aldrich) for protein removal and 300 μL of the solutions A, B, C, or D. Afterwards, all samples were vortexed, microcentrifuged (14,000 rpm for 20 min), diluted ten times with ultrapure water, filtered (0.2 μm regenerated cellulose (RC) filters; GE Healthcare Life Sciences, Little Chalfont, Buckinghamshire, UK), and injected (10 μL) into the column. The concentrations in the original samples were calculated as the intercepts with the X-axis of the linear regression of the peak heights (y-values) against the added concentrations (x-values).

### 2.5. Bread Production and Evaluation

Wheat breads (circa 500 g) with sourdough (21% on flour basis, m/m) were produced using the sourdoughs of the LAB- and AAB-initiated sourdough productions to evaluate the impact of these sourdoughs on the bread crumb volatile profiles, as described before [25]. Briefly, a dough was made of 342 g of wheat flour (same as above), 146 mL of ultrapure water (MilliQ), 3 g of dry commercial yeast (Instant Yeast Blue; Algist Bruggeman, Ghent, Belgium), 5 g of salt, and 72 g of sourdough in a bread machine (Home Bread Uno, Moulinex, Écully, France; dough preparation time of 136 min), which was baked for 63 min (program 3, browning scale 3). HS/SPME-GC-MS was applied on crust and crumb samples, as described above and previously [15]. The peak areas of the volatile organic compounds detected were normalized for subsequent PCA using the SPSS 20.0 software package for Windows (IBM), as described above.

Additionally, wheat breads with sourdough using the *G. oxydans* IMDO A845-initiated sourdoughs were evaluated as to their crust and crumb color, making use of a chromameter CR-400 (Minolta, Osaka, Japan). Reference wheat bread was prepared in the same way, using a 72-h fermented sourdough with a homofermentative LAB strain (*Companilactobacillus crustorum* LMG 23699 sourdough production LC400-Ctl1) [25]. The D65 settings were chosen for the illuminant and the CIE L* (lightness), a* (green-red component), and b* (blue-yellow component) color space values were measured. At least three different sites of either the crumb or crust were targeted and the resulting values from each site were the means of five measurements taken at different points in these sites. The values obtained were processed statistically using the SPSS 20.0 software package for Windows (IBM). The normality and homoscedasticity of the data were investigated using the Shapiro–Wilk test and the Levene test, respectively. A one-way analysis of variance (ANOVA) was performed when the values were normally distributed and had homogeneous variances. Otherwise, a Kruskal–Wallis test was performed. Significant differences among the samples were revealed using post-hoc methods, namely Tukey’s HSD or Dunett T3 in the case of non-homogeneity of variances, respectively.

To determine if *G. oxydans* IMDO A845-initiated sourdoughs caused the brown color of the breads made thereof, a sourdough sample (taken at 72 h) was heated for 15 min at 100 °C to simulate the heating during the bread baking process. The color of the heated sourdoughs and the baked breads was assessed visually.

## 3. Results

### 3.1. Sourdough pH and Total Titratable Acidity

**Figure 1:**
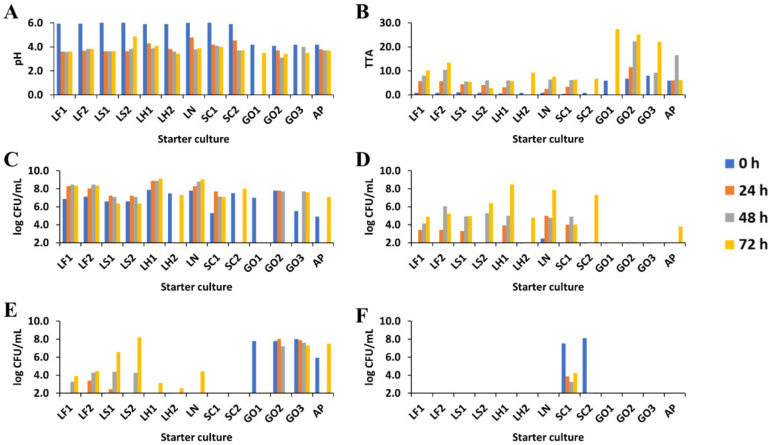
Evolution of pH (**A**), total titratable acidity (TTA) (**B**), and viable counts (**C**–**F**) of presumptive lactic acid bacteria (**C**), yeasts (**D**), acetic acid bacteria (**E**), and coagulase-negative staphylococci (**F**) during starter culture-initiated sourdough productions. The indication of the starter culture strains is as in Table 1.

The initial pH (5.90 ± 0.08) of the LAB- and CNS-initiated sourdough productions was higher than the initial pH (4.20 ± 0.05) of the AAB-initiated ones (Figure 1). The latter was due to the addition of acetic acid to the wheat flour–water mixtures at the start of the fermentation. On average, the pH decreased to 4.10 ± 0.46 (LAB, CNS) and 3.80 ± 0.07 (AAB) during the first 24 h of fermentation, and further down to 3.90 ± 0.45 (LAB, CNS) and 3.50 ± 0.13 (AAB) after 72 h of fermentation. The lowest pH of 3.45 (after 72 h of fermentation) was reached during the sourdough productions initiated with *G. oxydans* IMDO A845 (GO1, GO2, and GO3). The highest pH of 4.89 (after 72 h of fermentation) was reached during the *Latl. sakei* CTC 494-initiated sourdough production LS2.

The TTA values increased from an average of 0.8 ± 0.2 mL (LAB, CNS) and 6.7 ± 1.0 mL (AAB) at the start of fermentation to 7.4 ± 3.0 mL (LAB, CNS) and 20.3 ± 10.0 mL (AAB) after 72 h of fermentation (Figure 1). The higher initial TTA value in the case of the AAB-initiated sourdough productions was due to the addition of acetic acid to the wheat flour-water mixtures at the start of the fermentation.

### 3.2. Microbial Community Dynamics

#### 3.2.1. Microbial Enumerations

Average presumptive LAB counts of 6.7 ± 0.9 log (CFU/mL) were found on the mMRS-5 agar medium at the start of all the LAB-initiated sourdough productions (LF1, LF2, LS1, LS2, LH1, LH2, and LN; Figure 1). Presumptive LAB on the mMRS-5 agar medium were also found for all the AAB (AP, GO1, GO2, and GO3; average of 6.3 ± 1.3 log (CFU/mL)) and *St. carnosus* IMDO F4P1-initiated sourdough productions (SC1 and SC2; average of 4.3 ± 1.6 log (CFU/mL)); however, the AAB and CNS strains were able to grow on mMRS-5 as well. At the start of the SC1 and SC2 sourdough productions, average CNS counts of 7.8 ± 1.0 log (CFU/mL) were found on the MSA medium. Colonies on the mDMS agar medium were only found at the start of the AAB-initiated sourdough productions (AP, GO1, GO2, and GO3; on average 7.4 ± 1.0 log (CFU mL)). No or low (<2.5 log (CFU/mL)) yeast counts were found on the YPD agar medium.

After 72 h of fermentation, presumptive LAB counts of, on average, 7.7 ± 1.0 log (CFU/mL) were found on the mMRS-5 agar medium for all starter culture-initiated sourdough productions, except for the aerated (through the flour–water mixture) *G. oxydans* IMDO A845-initiated sourdough productions GO1 and GO2 that did not harbor LAB (Figure 1). For the GO1 and GO2 sourdough productions, no AAB counts could be retrieved from the respective agar media after 72 h of fermentation. Yet, the AAB counts of all AAB-initiated sourdough productions (AP, GO1, GO2, and GO3) increased throughout fermentation, except for the aerated (through the flour–water mixture) *G. oxydans* IMDO A845-initiated sourdough productions GO1 and GO2 that showed an increase in AAB counts until 48 h of fermentation. Throughout all starter culture-initiated sourdough productions, except for the *G. oxydans* IMDO A845-initiated ones (GO1, GO2, and GO3), variable yeast counts ranging from 3.8 to 7.2 log (CFU/mL) were found after 72 h of fermentation. No yeasts were found throughout the entire fermentation period of the *G. oxydans* IMDO A845-initiated sourdough productions GO1, GO2, and GO3. During the *St. carnosus* IMDO F4P1-initiated sourdough production SC1, the CNS counts decreased to 4.2 log (CFU/mL) after 72 h of fermentation, whereas no CNS counts were retrieved for *St. carnosus* IMDO F4P1-initiated sourdough production SC2 after 72 h of fermentation.

#### 3.2.2. Microbial Identifications

**Figure 2:**
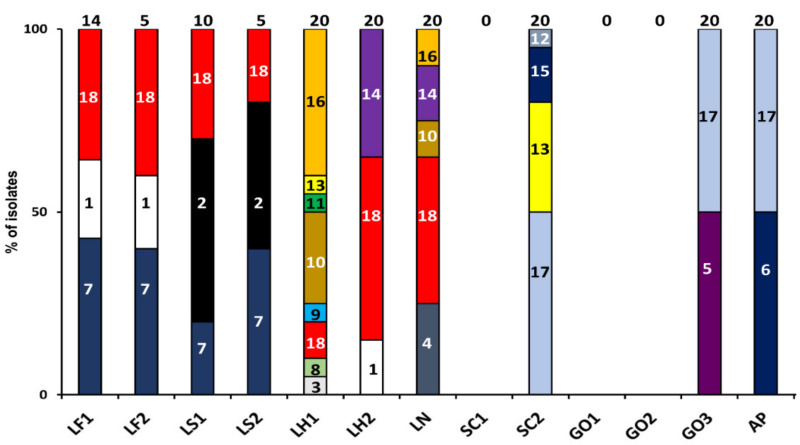
Culture-dependent microbial community dynamics and identifications of starter culture-initiated sourdough productions after 72 h of fermentation. The indication of the starter culture strains is as in Table 1. The total number of isolates identified is indicated on top of the bars. The numbers in the bars correspond with the following identities for the 16S rRNA gene: 1, *Limosilactobacillus fermentum* (100% identity; accession no. LC065036.1); 2, *Latilactobacillus sakei* (98% identity; accession no. NR_114915.1); 3, *Lentilactobacillus hilgardii* (100% identity; accession no. LC064898.1)*;* 4, *Liquorilactobacillus nagelii* (100% identity; accession no. LC383821.1); 5, *Gluconobacter oxydans* (100% identity; accession no. NR_118196.1); 6, *Acetobacter pasteurianus* (100% identity; accession no. NR_117258.1)*;* 7, *Acetobacter tropicalis* (100% identity; accession no. NR117258.1); 8, *Lactiplantibacillus plantarum* (100% identity; accession no. AB362982.1); 9, *Lactococcus lactis* (97% identity; accession no. KR604712.1); 10, *Companilactobacillus crustorum* (97% identity; accession no. JQ918151.1); 11, *Leuconostoc citreum* (98% identity; accession no. NR041727.1); 12, *Pediococcus acidilactici* (97% identity; accession no. LC071837.1); 13, *Pediococcus pentosaceus* (98% identity; accession no. LC071837.1); 14, *Weissella cibaria* (100% identity; accession no. LC096236.1); 15, *Weissella confusa* (100% identity; accession no. LC063164.1); 16, *Candida tropicalis* (99% identity; accession no. FJ483907.1); 17, *Saccharomyces cerevisiae* (99% identity; accession no. NG042623.1); 18, *Wickerhamomyces anomalus* (98% identity; accession no. EU057562.1).

**Figure 3:**
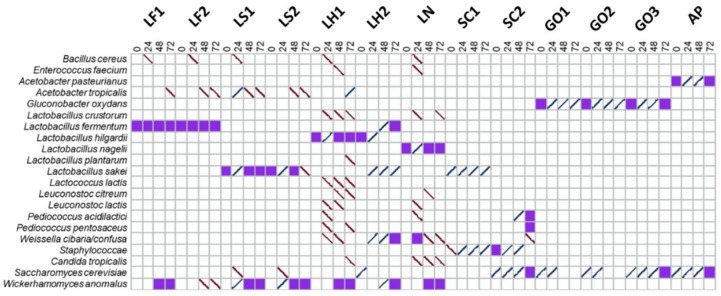
Microbial community dynamics during starter culture-initiated sourdough productions as determined by culture-dependent and culture-independent microbiological methods. The indication of the starter culture strains is as in Table 1. The species found both culture-dependently (molecular identification of picked isolates and PCR-DGGE of plate washes) and culture-independently (PCR-DGGE of fermentation samples) are indicated as purple squares, and those found only culture-dependently are indicated with downward stripes ( \ ), whereas those found only culture-independently are indicated with upward stripes ( / ).

In all sourdough productions, the starter culture species were present initially, as determined by both culture-dependent and culture-independent microbiological methods (Figure 2 and Figure 3).

Additionally, the yeast species *Pichia burtonii* (in the *Liml. fermentum* IMDO 222-initiated sourdough production LF1) and *Saccharomyces cerevisiae* (in the *St. carnosus* IMDO F4P1-initiated sourdough production SC2, the *G. oxydans* IMDO A845-initiated sourdough productions GO1, GO2, and GO3, and the *A. pasteurianus* IMDO 386B-initiated sourdough production AP) were found in the initial wheat flour–water mixtures, as determined culture-independently (Figure 3).

Upon further fermentation, culture-dependent and culture-independent microbiological analyses showed the presence of *Liml. fermentum*, *Acetobacter tropicalis*, and *Wickerhamomyces anomalus* during the *Liml. fermentum* IMDO 222-initiated sourdough productions LF1 and LF2, from 48 to 72 h of fermentation (Figure 2 and Figure 3). Similarly, the *Latl. sakei* CTC 494-initiated sourdough productions LS1 and LS2 were characterized by *Latl. sakei*, *A*. *tropicalis*, and *W*. *anomalus* from 48 h onwards. In contrast, the sourdough productions initiated with *Lenl. hilgardii* IMDO 2M0G15 (LH1 and LH2) and *Liql*. *nagelii* IMDO 2M48G6 (LN) were not characterized by their starter culture species inoculated, after 72 h of fermentation, but harbored a complex microbial consortium, including different LAB species, *Candida tropicalis*, and *W. anomalus.*

The sourdough productions initiated with the AAB strains *A. pasteurianus* IMDO 386B (AP) and *G. oxydans* IMDO A845 (GO1, GO2, and GO3) were characterized by these species, together with *S. cerevisiae*, as shown culture-dependently and/or culture-independently (Figure 2 and Figure 3). The presence of *St. carnosus* in the *St. carnosus* IMDO F4P1-initiated sourdough production SC1 could only be demonstrated using plate washes. In contrast, this species was found in both plate washes and through direct 16S rRNA-PCR-DGGE community profiling in the sourdough samples of the *St. carnosus* IMDO F4P1-initiated sourdough production SC2. In the latter case, it occurred together with *Pediococcus* spp. and *S. cerevisiae*, among others.

### 3.3. Substrate Consumption and Metabolite Production Kinetics

#### 3.3.1. Substrate Consumption Kinetics

**Figure 4:**
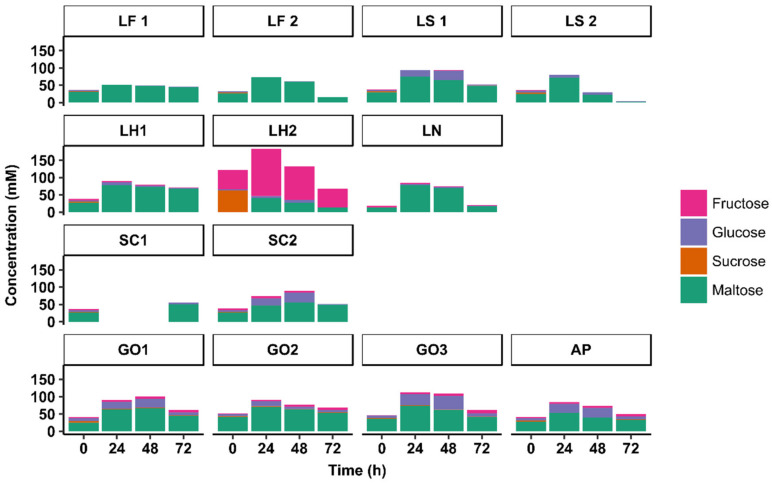
Carbohydrate concentrations during starter culture-initiated sourdough productions. The indication of the starter culture strains is as in Table 1.

Initially, in all sourdough productions, except for the *Lenl. hilgardii* IMDO 2M0G15-initiated sourdough production LH2, maltose was the major carbohydrate present, with an average concentration of 26.0 ± 9.3 mM, followed by glucose (4.8 ± 2.3 mM), fructose (2.5 ± 1.3 mM), and sucrose (2.6 ± 0.8 mM) (Figure 4). In the *Lenl. hilgardii* IMDO 2M0G15-initiated sourdough production LH2, higher initial concentrations of sucrose (63.2 mM) and fructose (55.2 mM) were found due to the addition of the former carbohydrate at the start, whereas the concentrations of the other carbohydrates were as the average values mentioned above.

Over the 72-h course of all sourdough productions, none of the starter culture strains examined depleted the concentrations of maltose, albeit a wide range of residual maltose concentrations was found (from 2.2 mM (*Latl. sakei* CTC 494-initiated sourdough production LS2) to 67.6 mM (*Lenl. hilgardii* IMDO 2M0G15-initiated sourdough production LH1)). Low concentrations of glucose were found at the end of all sourdough productions. During the AAB-initiated sourdough productions (AP, GO1, GO2, and GO3), an initial increase in the glucose concentrations took place, presumably due to the activation of amylases by the low initial pH upon the addition of acetic acid to the wheat flour–water mixtures at the start of the fermentation. This was also the case for the sourdough productions LS1 (initiated with *Latl. sakei* CTC 494) and SC2 (initiated with *St. carnosus* IMDO F4P1), presumably due to a low glucose consumption. The concentrations of fructose were elevated during the *Lenl. hilgardii* IMDO 2M0G15-initiated sourdough production LH2, presumably due to the addition of sucrose at the start of the fermentation and the preferred consumption of glucose. The concentrations of fructose increased during the AAB-initiated sourdough productions (AP, GO1, GO2, and GO3) too. Sucrose was depleted in all sourdough productions after 24 h of fermentation, except for the AAB-initiated ones (AP, GO1, GO2, and GO3), during which low residual sucrose concentrations were found after 72 h of fermentation (<2 mM).

#### 3.3.2. Metabolite Production Kinetics

**Figure 5:**
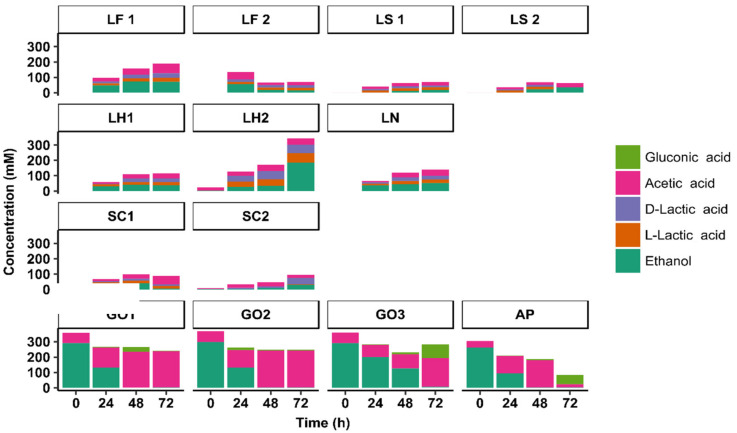
Concentrations of acetic acid, ethanol, gluconic acid, and lactic acid (D and L) during starter culture-initiated sourdough productions. The indication of the starter culture strains is as in Table 1.

The concentrations of L-lactic acid and D-lactic acid increased during all LAB-initiated sourdough productions (LF1, LF2, LS1, LS2, LH1, LH2, and LN) as well as during the *St. carnosus* IMDO F4P1-initiated sourdough productions SC1 and SC2 (Figure 5). No lactic acid was found in the AAB-initiated sourdough productions (AP, GO1, GO2, and GO3). The highest concentrations of lactic acid were found during the *Lenl. hilgardii* IMDO 2M0G15-initiated sourdough production LH2. Throughout the *Liml. fermentum* IMDO 222-initiated sourdough productions LF1 and LF2, the fractions of L-lactic acid were between 45 and 51% of the total lactic acid concentrations. For the *Latl. sakei* CTC 494-initiated sourdough productions LS1 and LS2, these fractions were between 57 and 63%, whereas for the *Lenl. hilgardii* IMDO 2M0G15- (LH1 and LH2) and *Liql. nagelii* IMDO 2M48G6-initiated sourdough productions (LN), they ranged from 44 to 52%, except for the sample taken after 24 h during the *Lenl. hilgardii* IMDO 2M0G15-initiated sourdough production LH1 (72%). The values found for the *St. carnosus* IMDO F4P1-initiated sourdough productions SC1 and SC2 differed, being 61% and <10%, respectively, indicating that different microbial communities prevailed during these fermentation processes.

Low acetic acid concentrations (<6 mM) were found at the start of all sourdough productions, except for the AAB-initiated ones (AP, GO1, GO2, and GO3), for which acetic acid was added to the wheat flour–water mixtures at the start of the fermentation to reach an average concentration of 63 ± 28 mM (Figure 5). The concentrations of acetic acid increased throughout all the LAB- (LF1, LF2, LS1, LS2, LH1, LH2, and LN) and *St. carnosus* IMDO F4P1-initiated sourdough productions (SC1 and SC2) to an average of 36 ± 28 mM. During the *G. oxydans* IMDO A845-initiated sourdough productions (GO1, GO2, and GO3), the acetic acid concentrations increased throughout the entire 72-h fermentation processes. Higher maximal concentrations were found for the *G. oxydans* IMDO A845-initiated sourdough productions GO1 and GO2 aerated through the flour–water mixtures (240 mM) than for that aerated through the fermenter headspace (GO3, 190 mM). In contrast, the concentration of acetic acid decreased after 48 h of fermentation during the *A. pasteurianus* IMDO 386B-initiated sourdough production AP.

As for acetic acid, high concentrations of ethanol were found at the start of all AAB-initiated sourdough productions (AP, GO1, GO2, and GO3), but not at the start of all LAB- (LF1, LF2, LS1, LS2, LH1, LH2, and LN) and *St. carnosus* IMDO F4P1-initiated sourdough productions (SC1 and SC2) (Figure 5). Elevated ethanol concentrations were found after 72 h of fermentation during the *Lenl. hilgardii* IMDO 2M0G15-initiated sourdough production LH2, presumably due to high carbohydrate consumption by the yeast species. During the AAB-initiated sourdough productions (AP, GO1, GO2, and GO3), the ethanol concentration decreased below 4 mM after 72 h of fermentation, due to its oxidation into acetic acid.

**Figure 6:**
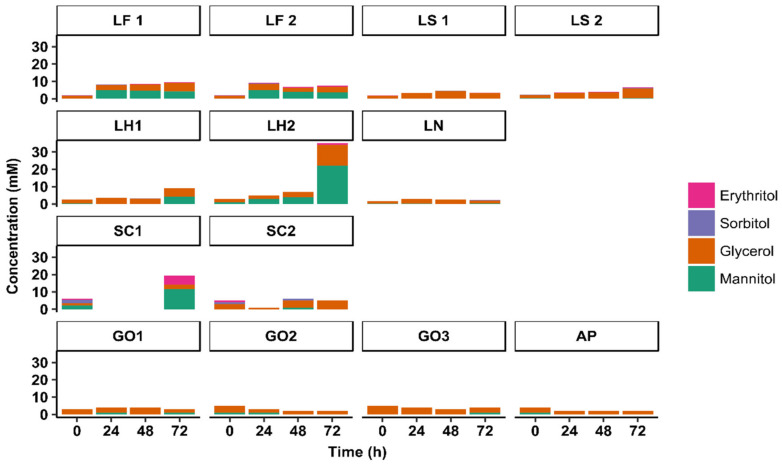
Concentrations of sugar alcohols during starter culture-initiated sourdough productions. The indication of the starter culture strains is as in Table 1.

Low concentrations of the sugar alcohols erythritol, glycerol, mannitol, and sorbitol were found at the start of all sourdough productions, with average values of 0.2 ± 0.4 mM, 2.3 ± 1.1 mM, 0.5 ± 0.6 mM, and 0.2 ± 0.5 mM, respectively (Figure 6). The concentrations of sorbitol did not change during these production processes, whereas the concentrations of the other sugar alcohols changed only during dedicated ones. For instance, the glycerol concentration increased during the *Lenl. hilgardii* IMDO 2M0G15-initiated sourdough production LH2, with a final concentration of 12.2 ± 0.1 mM, and that of erythritol increased during the *St. carnosus* IMDO F4P1-initiated sourdough production SC1, with a final concentration of 5.3 ± 0.3 mM.

Mannitol was produced during the sourdough productions initiated with *Liml. fermentum* IMDO 222 (LF1 and LF2) as well as during the *St. carnosus* IMDO F4P1-initiated sourdough production SC1, with concentrations of 4 mM and 12 mM, respectively, after 72 h of fermentation. High mannitol concentrations were found during the sucrose-added *Lenl. hilgardii* IMDO 2M0G15-initiated sourdough production LH2 (22 mM) in contrast to that without sucrose supplementation (LH1, 4 mM). Elevated concentrations of gluconic acid were found after 72 h of fermentation during the *A. pasteurianus* IMDO 386B-initiated sourdough production AP (61 ± 2 mM) and the *G. oxydans* IMDO A845-initiated sourdough production GO3 (89 ± 2 mM) that was aerated only through the fermenter headspace (Figure 5). Lower concentrations of gluconic acid (<10 mM) were found at the end of the 72-h *G. oxydans* IMDO A845-initiated sourdough productions GO1 and GO2 that were aerated throughout the wheat flour–water mixtures. During these sourdough productions, the gluconic acid concentrations decreased after an initial increase. Low concentrations of 2-ketogluconic acid (3.4 ± 0.1 mM) were found at the end of the *G. oxydans* IMDO A845-initiated sourdough production GO3 that was aerated only through the fermenter headspace. In contrast, its concentrations during the other AAB-initiated sourdough productions (AP, GO1, and GO2) were below 1 mM. Furthermore, 5-ketogluconic acid was not found during any of the AAB-initiated sourdough productions (AP, GO1, GO2, and GO3).

**Figure 7:**
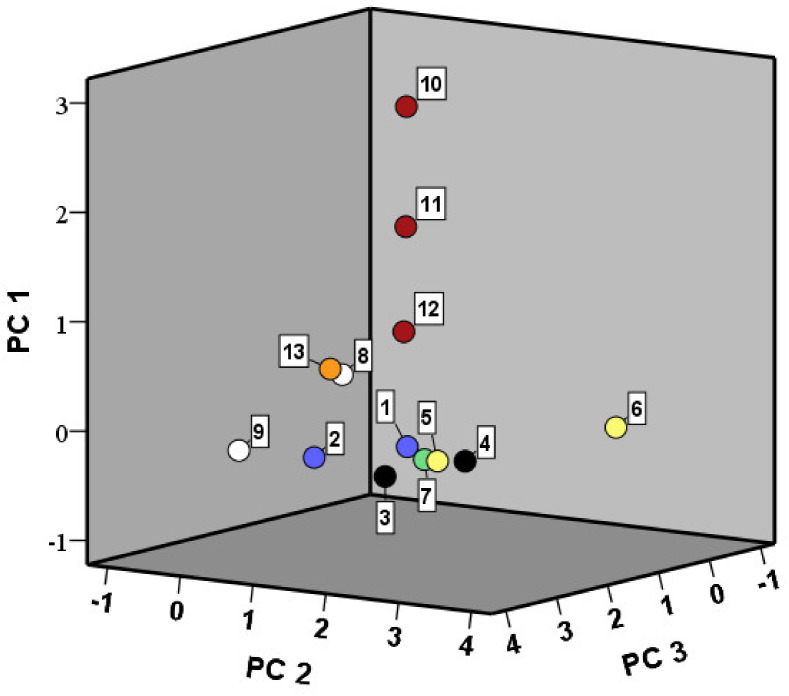
Principal component analysis (PCA) of the normalized peak areas of the volatile organic compounds present in the starter culture-initiated sourdough productions after 72 h of fermentation. The numbers indicate the different starter cultures used: 1, *Limosilactobacillus fermentum* IMDO 222 (LF1); 2, *Liml. fermentum* IMDO 222 (LF2); 3, *Latilactobacillus sakei* CTC 494 (LS1); 4, *Latl. sakei* CTC 494 (LS2); 5, *Lentilactobacillus hilgardii* IMDO 2M0G15 (LH1); 6, *Lenl. hilgardii* IMDO 2M0G15 (LH2); 7, *Liquorilactobacillus nagelii* IMDO 2M48G6 (LN); 8, *Staphylococcus carnosus* IMDO F4P1 (SC1); 9, *St. carnosus* IMDO F4P1 (SC2); 10, *Gluconobacter oxydans* IMDO A845 (GO1); 11, *G. oxydans* IMDO A845 (GO2); 12, *G. oxydans* IMDO A845 (GO3); 13, *Acetobacter pasteurianus* IMDO 386B (AP).

A total of 117 volatile organic compounds were identified through HS/SPME-GC-MS in all sourdoughs together. Through PCA, three PCs were taken into account, covering 54% of the total variance of the data (Figure 7; Table 2). PC1 (24% of the total variance) was characterized by positive factor loadings for aldehydes. PC2 (16%) was characterized by positive factor loadings for mainly esters. PC3 (14%) was characterized by positive factor loadings for alcohols. A three-dimensional score-plot of the sourdoughs revealed the production of specific volatile organic compounds in some cases, due to the application of different starter culture strains. For instance, GO1 and GO2 scored high on PC1, whereas LH2 scored high on PC2.

### 3.4. Bread Production

#### 3.4.1. Volatile Organic Compounds

**Figure 8:**
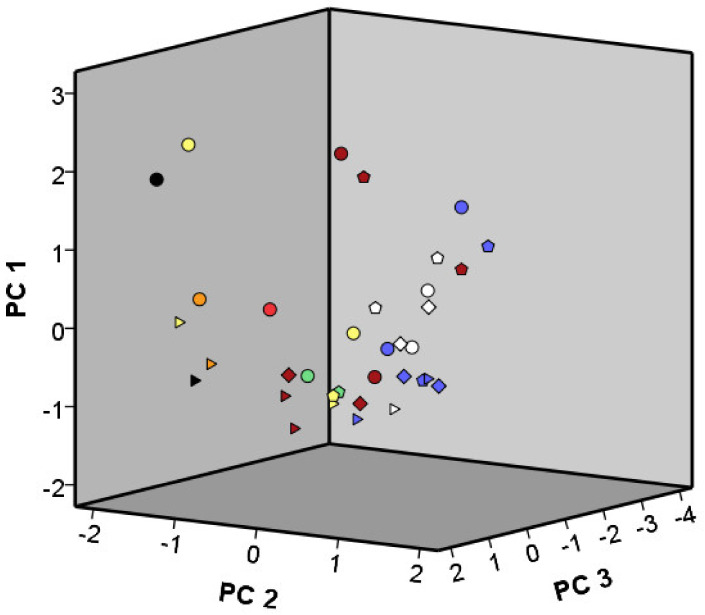
Principal component analysis (PCA) of the normalized peak areas of the volatile organic compounds present in the crumbs of wheat breads made with starter culture-initiated sourdoughs. The starter cultures are indicated with colored symbols: *Acetobacter pasteurianus* IMDO 386B, orange; *Limosilactobacillus fermentum* IMDO 222, blue; *Gluconobacter oxydans* IMDO A845 (aerated through the flour–water mixture (GO1 and GO2), dark red; aerated through the fermenter headspace (GO3), red); *Lentilactobacillus hilgardii* IMDO 2M0G15, yellow; *Liquorilactobacillus nagelii* IMDO 2M48G6, green; *Latilactobacillus sakei* CTC 494, white; *Staphylococcus carnosus* IMDO F4P1, black. The symbol shapes indicate the fermentation time of the sourdough productions: triangles, 0 h; diamonds, 24 h; pentagons, 48 h; and circles, 72 h.

Three PCs for a PCA of the bread crumb volatile organic compounds explained 40% of the total variance and illustrated the impact of the different starter culture strains tested on the breads made with the concomitant sourdoughs (Figure 8; Table 3). Yet, the effect was much less apparent than for the sourdoughs themselves. Breads made with sourdoughs initiated with *Lenl. hilgardii* IMDO 2M0G15 (LH1 and LH2) and *St. carnosus* IMDO F4P1 (SC1 and SC2) scored generally high on PC1 (15% of the total variance), whereas breads made with sourdoughs fermented by *Liml. fermentum* IMDO 222 (LF1 and LF2) and *Latl. sakei* CTC 494 (LS1 and LS2) scored mostly high on PC2 (15%). Those fermented by *G. oxydans* IMDO A845 (GO1, GO2, and GO3) scored low on PC3 (10%). However, breads produced with 48- and 72-h sourdoughs fermented by *G. oxydans* IMDO A845 aerated through the flour–water mixture (GO1 and GO2) had a donut aroma, which was not detected in any other bread produced.

#### 3.4.2. Color

**Figure 9:**
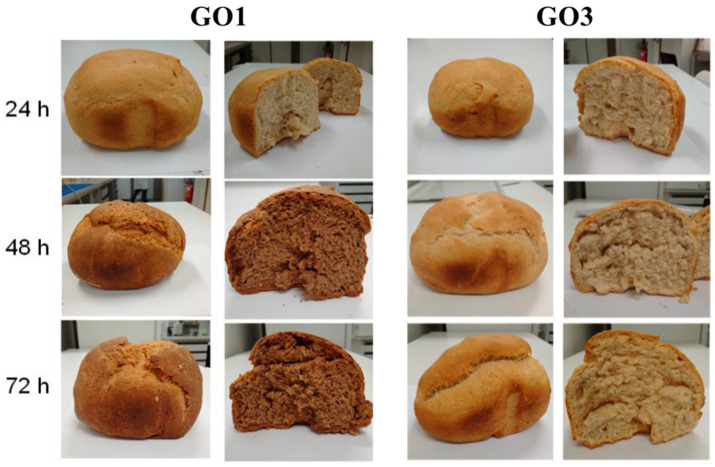
Wheat breads made with sourdoughs obtained through *Gluconobacter oxydans* IMDO A845 (GO1)-initiated fermentations (aeration through the wheat flour–water mixture) and *G. oxydans* IMDO A845 (GO3)-initiated fermentations (aeration through the fermenter headspace) at three different sampling points.

An increasing fermentation time of the *G. oxydans* IMDO A845-initiated sourdough productions GO1 and GO2 that were aerated through the flour–water mixture led to significantly darker brown-colored wheat breads compared with the reference wheat breads, as indicated by the decreasing L* (lightness) and increasing a* (red-green color) values for the bread crumb and crust colors (Table 4; Figure 9). This was not the case for the wheat breads made with the *G. oxydans* IMDO A845-initiated sourdough production, whose fermentation was aerated through the fermenter headspace (GO3; Figure 9).

Heating of a sourdough sample of a *G. oxydans* IMDO A845-initiated sourdough production (GO2) after 72 h of fermentation resulted in a brown pigmentation of the sourdough.

## 4. Discussion

The application of an appropriate, competitive starter culture is of utmost importance to produce liquid, Type II/2 sourdoughs [3]. Besides fast acidification, a functional starter culture also expresses additional features, such as improved flavor and texture [24]. Therefore, functional starter culture strains, to be used, have to be highly adapted to the cereal matrix [2]. Consequently, in the past, mainly sourdough-specific strains have been examined regarding their metabolic properties of interest to produce sourdoughs, which are related to their competitiveness in this matrix [3]. Alternatively, both LAB and non-LAB starter culture strains, originating from non-cereal matrices, might possess interesting metabolic pathways not commonly found in sourdough-specific bacteria due to the adaptation to the fermented food matrix that these starter cultures are isolated from. These pathways might lead to the in situ expression of novel, desirable properties, in turn leading to innovative sourdoughs.

To this end, several starter culture strains possessing interesting pathways and isolated from different fermenting food matrices were tested as to their prevalence, and their contribution to the productions was tested [3,16,19,67,68,69]. *Limosilactobacillus fermentum* IMDO 222, previously isolated from a fermenting cocoa pulp-bean mass, was able to prevail during cocoa fermentation processes when applied as a starter culture [70]. Similarly, as shown in the present study, this strain prevailed during Type II/2 sourdough productions, which might be related to its enhanced competitiveness when growing on maltose (thanks to the presence of a maltose phosphorylase) as an energy source and using fructose as an alternative external electron acceptor [71]. Furthermore, it produces buttery flavored compounds, in accordance with its genetic potential [69,71]. This indicated the potential of this strain and, most probably, other *Liml. fermentum* strains as starter cultures for a variety of food matrices [28,70,72]. Likewise, *Latl. sakei* CTC 494, a sausage starter culture strain with a rather weak acidification potential [42], was able to prevail during the Type II/2 sourdough productions of the current study, likely due to its enhanced competitiveness [42,73,74]. The species *Latl. sakei* participates in multiple sourdough fermentation processes, in particular those with an elevated pH [3,11,67]. In contrast, the strains of the species *Lenl. hilgardii* and *Liql. nagelii*, LAB species that are not commonly found in sourdoughs, were not capable of prevailing during the sourdough productions performed. This might be due to both species- and strain-specific adaptations to the matrices they originate from (in casu water kefir) which are not exchangeable with the sourdough matrix. Indeed, water kefir is a unique fermented ecosystem, harboring a consortium of dedicated LAB species (besides *Lenl. hilgardii* and *Liql. nagelii*, also *Lacticaseibacillus paracasei*, a LAB species similar to *Liquorilactobacillus hordei/mali*, and *Oenococcus sicerae*, bifidobacterial species (*Bifidobacterium aquikefiri*), and yeast species (*S. cerevisiae* and *Dekkera bruxellensis*) [38,39,44,50,51,75,76,77]. Moreover, LAB species frequently found in cereal fermentations are often animal-associated (e.g., gut microbiota; *Liml. fermentum*, *Lb. reuteri*, *Latl. sakei*, etc.) or vegetable fermentation process-associated (e.g., *Lb. plantarum*) [78]. In contrast, *Lenl. hilgardii* and *Liql. nagelii* are most commonly associated with alcoholic fermentation processes, such as stuck wine and water kefir fermentations; in the latter case, sucrose is the main carbohydrate source, which is necessary for both energy production and water kefir grain growth [39,50,51,79]. This might indicate that these species, regardless of the strains, are less suitable as starter cultures for sourdough productions, which are characterized by having maltose as the driving energy source. Indeed, the addition of sucrose influenced the growth and metabolite production during the *Lenl. hilgardii* IMDO 2M0G15-initiated sourdough production LH2, as indicated by the elevated concentrations of ethanol, glycerol, lactic acid, and mannitol. The latter compound indicated the use of fructose, originating from the sucrose added, as an alternative external electron acceptor.

Besides lactobacilli, CNS, in particular *St. carnosus*, belong to the microbial groups determining sausage fermentation processes [61,80]. They contribute to flavor and color development during meat fermentation [61]. Staphylococci are common members of flour; however, they are not competitive to survive during sourdough productions [3]. Indeed, as shown in the present study, even if inoculated into the flour–water mixture and with the addition of salt that favors their growth, they did not prevail during the sourdough production process.

Finally, strains of AAB species isolated from fermenting cocoa pulp-bean mass were tested. *Acetobacter pasteurianus* prevails during the aerobic phase of cocoa pulp-bean mass fermentations, oxidizing the ethanol produced by the yeasts during the preceding anaerobic phase to acetic acid [35,40,46,47]. In the presence of carbohydrate excesses during the aerobic phase, *G. oxydans* oxidizes glucose to gluconic acid [65,81]. Under the conditions of the present study, the *A. pasteurianus* and *G. oxydans* strains tested were able to prevail during the fermentations of the sourdough productions initiated with them. The substrate consumption and metabolite production kinetics during those fermentations correlated with their carbohydrate consumption patterns, whereby fructose was not consumed. Instead, the concentrations of fructose increased, likely due to its continuous release from sucrose through invertase activity or by oxidation of mannitol by a polyol oxidoreductase [37,41]. Furthermore, the high concentrations of acetic acid and gluconic acid showed the oxidation of ethanol and glucose, respectively [37,55]. The increase, followed by a decrease, in the acetic acid concentrations in the case of the *A. pasteurianus* IMDO 386B-initiated sourdough productions, but not in those initiated with *G. oxydans* IMDO A845, corresponded with the capacity for overoxidation of acetic acid to carbon dioxide and water of the former strain [37]. This capacity is generally absent in strains of *Gluconobacter* species [81]. Yet, aeration through the flour–water mixture influenced the metabolite production kinetics of the *G. oxydans* IMDO A845-initiated sourdough productions, leading to lower concentrations of gluconic acid and 2-ketogluconic acid and suggesting a further conversion of the former compound under these aeration conditions.

Of particular importance, aeration through the flour–water mixture of the *G. oxydans* IMDO A845-initiated sourdough productions influenced the color of the sourdoughs and wheat breads made thereof. It has previously been reported that the use of a *Gluconobacter albinus* strain leads to darker colored breads [57]. In the latter study, the brown color has been ascribed to the addition of molasses as a carbohydrate source to the buckwheat flour–water mixture used. However, as no molasses were added during the sourdough productions of the present study, the low pigmentation of the sourdough during fermentation and the high pigmentation of the sourdough upon heating and of the baked breads could be ascribed to the growth of and gluconic acid production and conversion by *G. oxydans*. Indeed, *Gluconobacter* species can produce dihydroxyacetone and (keto)gluconic acids [82]. The formation of the latter compound was shown during the present study but was not measured in the study of Ua-Arak et al. [57]. These heat-labile compounds can take part in Maillard-type reactions upon heating [83]. Furthermore, the enhanced color formation upon enhanced aeration might be due to the oxygen-dependent production of dihydroxyacetone, albeit that the latter compound was not measured in the present study [84,85]. Yet, the production of dihydroxyacetone could be assumed, given the decreased bacterial counts in these sourdoughs due to its toxicity. Consequently, the *G. oxydans* strain tested in the present study showed potential for the production of breads with sourdough, in particular brown wheat-based breads [68].

## 5. Conclusions

In conclusion, based on their prevailing growth and metabolite production kinetics, *Liml. fermentum* IMDO 222, *Latl. sakei* CTC 494, *A. pasteurianus* IMDO 386B, and *G. oxydans* IMDO A845 are candidate starter cultures for Type II/2 sourdough production processes. In particular, *G. oxydans* IMDO A845 had an interesting impact on the volatile organic compound production and color formation associated with its sourdoughs and the wheat-based breads produced thereof.

## Figures and Tables

**Table 1:** Bacterial strains selected as to their sourdough starter culture potential, source of isolation of the starters, ingredients used, and specific fermentation conditions applied for starter culture-initiated, liquid (Type II/2) sourdough productions.

Starter Culture Strain (References)	Source of Isolation	Sourdough Production Code	Ingredient Supplementation of the Flour–Water Mixture and Specific Fermentation Conditions
**Lactic acid bacteria (LAB)**			
*Limosilactobacillus fermentum* IMDO 222 [35,63]	Fermenting cocoa pulp-bean mass	LF1, LF2	None
*Latilactobacillus sakei* CTC 494 [36,42]	Fermented sausage	LS1, LS2	None
*Lentilactobacillus hilgardii* IMDO 2M0G15 [38,39]	Water kefir	LH1, LH2	None (LH1) or sucrose (40 g/L, LH2) *^a^*
*Liquorilactobacillus nagelii* IMDO 2M48G6 [38,39]	Water kefir	LN	None
**Coagulase-negative staphylococci (CNS)**			
*Staphylococcus carnosus* IMDO F4P1 [43]	Fermented sausage	SC1, SC2	None (SC1) or NaCl (40 g/L, SC2) *^b^*
**Acetic acid bacteria (AAB)**			
*Gluconobacter oxydans* IMDO A845 [35,47]	Fermenting cocoa pulp-bean mass	GO1, GO2, GO3	Acetic acid (3 g/L), ethanol (10 g/L), and aeration (5 L/min) through the wheat flour-water mixture (GO1, GO2) or the fermenter headspace (GO3) *^c^*
*Acetobacter pasteurianus* IMDO 386B [35,37,40,41]	Fermenting cocoa pulp-bean mass	AP	Acetic acid (3 g/L), ethanol (10 g/L), and aeration (5 L/min) through the wheat flour-water mixture *^c^*

*^a^* Sucrose was added to favor the growth of the LAB strain. *^b^* NaCl was added to favor the growth of the CNS strain. *^c^* Acetic acid and ethanol were added and aeration was provided to favor the growth of the AAB strain.

**Table 2:** Volatile organic compounds identified through solid-phase microextraction GC coupled to mass spectrometry (SPME-GC-MS) in starter culture-initiated sourdough productions according to the three principal components (PC) obtained after principle component analysis of all data.

PC1	PC2	PC3
1,3-Octadiene	2,4-Heptadienal (E,E)	1-Decanol
1-Nitro-hexane	2-Ethylhexanol	1-Ethyl-1-methylcyclopentane
1-Undecanol	2-Octanol	1-Heptanol
2,4-Decadienal (E,E)	3-Nonen-1-ol	1-Nonanol
2,4-Nonadienal (E,E)	3-Nonenyl acetate	1-Octanol
2,5-Octanedione	4-Methyl-2-pentanol	2-Nonanone
2-Cyclopentene-1,4-dione	Ethyl acetate	2-Nonen-1-ol
2-Decenal, (E)	Ethyldecanoate	2-Octanone
2-Ethyl-3-methyl-pyrazine	Heptyl acetate	3,5-Octadien-2-ol
2-Furanmethanol	Hexyl acetate	3-Methylbutanoic acid
2-Methoxyphenol	Hexyl-2-methoxyacetate	3-Octanone
2-Methyl-5-(1-methylethenyl)-cyclohexanol	Isobutyloctanoate	Butanoic acid
2-Nonenal (E)	Isoledene	Dodecane
2-Octenal (E)	Methylpyrazine	Ethyl heptanoate
2-Pentylfuran	Octyl acetate	
2-Propylphenol	Pentyl acetate	
2-Undecenal	Vinylhexanoate	
3-Methyl-6-propyl-phenol		
4-Oxononanal		
5-Ethylcyclopent-1-enecarboxaldehyde		
5-Methyl-2-furancarboxaldehyde		
6-Methyl-5-hepten-2-one		
Acetic acid		
Decanal		
Ethylpyrazine		
Hexanal		
Nonanal		
Octanal		
Pentanal		
Precocene 1		
Tetradecane		
Toluene		
Trimethylsilyl-di(trimethylsiloxy)-silane		

**Table 3:** Volatile organic compounds identified through SMPE-GC-MS in the crumbs of wheat breads made with starter culture-initiated sourdoughs according to the three principal components (PC) obtained after principle component analysis of all data.

PC1	PC2	PC3
1-Decanol	1-Octen-3-one	2,4-Decadien-1-ol (E,Z)
1-Heptanol	2,3-Pentanedione	3,5-Octadien-2-one
1-Octanol	2,3-Butanedione	3-Methyl-1-butanol
1-Octen-3-ol	2-Methylnaphthalene	3-Methylthiopropanol
2,3-Octanedione	Butanoic acid	3-Nonen-1-ol (Z)
2,5-Octanedione	Dodecane	3-Nonenyl acetate
2-Heptanal (Z)	Ethanol	Ethyl decanoate
2-Octanone	Nonanoic acid	Hexyl acetate
2-Octenal (E)	Naphthalene	Vinyl caproate
2-Pentylfuran	Octanoic acid	
3-Methyl-6-propyl-phenol		
5-Methyl-2-furancarboxaldehyde		
Benzaldehyde		
Ethyl octanoate		
Hexanal		
Octanal		

**Table 4:** Colorimetric evaluation, using L* (lightness), a* (green-red component), and b* (blue-yellow component) color values of the crumb and crust of wheat breads made with *Gluconobacter oxydans* IMDO A845-initiated sourdoughs (unfermented or fermented for 72 h) and aerated through the wheat flour–water mixture (sparged) or through the fermenter headspace (aerated). Statistically significant differences among samples within each column are indicated by a letter in superscript.

Sourdough Used for Wheat Bread Production	Crumb Color			Crust Color		
	L *	a *	b *	L *	a *	b *
Reference	73.1 ± 5.3 ^a^	0.3 ± 0.3 ^a^	16.6 ± 1.1	60.0±4.3 ^a,d^	11.7 ± 2.1 ^a^	32.5 ± 2.2 ^a,b^
Sparged, 0 h	72.5 ± 4.2 ^a^	0.1 ± 0.1 ^a^	15.9 ± 1.2	60.8±5.8 ^a,d^	11.5 ± 3.4 ^a^	33.1 ± 1.5 ^a,b^
Sparged, 24 h	72.3 ± 4.2 ^a^	0.2 ± 0.2 ^a^	16.5 ± 2.1	59.8±3.0 ^a,c,d^	12.3 ± 2.6 ^a^	35.2 ± 1.6 ^a,b^
Sparged, 48 h	45.5 ± 3.6 ^b^	9.1 ± 0.6 ^b^	17.6 ± 1.3	44.9±3.5 ^b,d^	14.1 ± 0.7 ^a^	24.4 ± 0.4 ^c,d^
Sparged, 72 h	51.3 ± 1.2 ^b^	8.4 ± 0.3 ^b^	17.6 ± 0.8	44.6±2.0 ^b,d^	14.0 ± 1.4 ^a^	23.2 ± 1.9 ^c^
Aerated, 72 h	72.9 ± 1.5 ^a^	0.7 ± 1.1 ^a,c^	16.4 ± 2.5	62.8±2.7 ^a,d^	12.1 ± 1.8 ^a^	36.9 ± 2.7 ^a^

## References

[1] De Vuyst L., Harth H., van Kerrebroeck S., Leroy F. (2016). Yeast diversity of sourdoughs and associated metabolic properties and functionalities. Int. J. Food Microbiol..

[2] De Vuyst L., van Kerrebroeck S., Harth H., Huys G., Daniel H.M., Weckx S. (2014). Microbial ecology of sourdough fermentations: Diverse or uniform?. Food Microbiol..

[3] De Vuyst L., van Kerrebroeck S., Leroy F. (2017). Microbial ecology and process technology of sourdough fermentation. Adv. Appl. Microbiol..

[4] De Vuyst L., Vrancken G., Ravyts F., Rimaux T., Weckx S. (2009). Biodiversity, ecological determinants, and metabolic exploitation of sourdough microbiota. Food Microbiol..

[5] Gänzle M.G., Ripari V. (2016). Composition and function of sourdough microbiota: From ecological theory to bread quality. Int. J. Food Microbiol..

[6] Gobbetti M., de Angelis M., Di Cagno R., Calasso M., Archetti G., Rizzello C.G. (2019). Novel insights on the functional/nutritional features of the sourdough fermentation. Int. J. Food Microbiol..

[7] Gobbetti M., Minervini F., Pontonio E., Di Cagno R., de Angelis M. (2016). Drivers for the establishment and composition of the sourdough lactic acid bacteria biota. Int. J. Food Microbiol..

[8] Gobbetti M., Rizzello C.G., Di Cagno R., de Angelis M. (2014). How the sourdough may affect the functional features of leavened baked goods. Food Microbiol..

[9] Minervini F., de Angelis M., Di Cagno R., Gobbetti M. (2014). Ecological parameters influencing microbial diversity and stability of traditional sourdough. Int. J. Food Microbiol..

[10] Settanni L., Speranza B., Bevilacqua A., Corbo M.R., Sinigaglia M. (2017). Sourdough and cereal-based foods: Traditional and innovative products. Starter Cultures in Food Production.

[11] Van Kerrebroeck S., Maes D., de Vuyst L. (2017). Sourdoughs as a function of their species diversity and process conditions, a meta-analysis. Trends Food Sci. Technol..

[12] Minervini F., Lattanzi A., de Angelis M., Celano G., Gobbetti M. (2015). House microbiotas as sources of lactic acid bacteria and yeasts in traditional Italian sourdoughs. Food Microbiol..

[13] Ripari V., Gänzle M.G., Berardi E. (2016). Evolution of sourdough microbiota in sponthaneous sourdoughs started with different plant materials. Int. J. Food Microbiol..

[14] Scheirlinck I., van der Meulen R., de Vuyst L., Vandamme P., Huys G. (2009). Molecular source tracking of predominant lactic acid bacteria in traditional Belgian sourdoughs and their production environments. J. Appl. Microbiol..

[15] Harth H., van Kerrebroeck S., de Vuyst L. (2016). Community dynamics and metabolite target analysis of spontaneous, backslopped barley sourdough fermentations under laboratory and bakery conditions. Int. J. Food Microbiol..

[16] Van der Meulen R., Scheirlinck I., van Schoor A., Huys G., Vancanneyt M., Vandamme P., de Vuyst L. (2007). Population dynamics and metabolite target analysis of lactic acid bacteria during laboratory fermentations of wheat and spelt sourdoughs. Appl. Environ. Microbiol..

[17] Van Kerrebroeck S., Bastos F.C., Harth H., de Vuyst L. (2016). A low pH does not determine the community dynamics of spontaneously developed backslopped liquid wheat sourdoughs but does influence their metabolite kinetics. Int. J. Food Microbiol..

[18] Weckx S., van der Meulen R., Allemeersch J., Huys G., Vandamme P., van Hummelen P., de Vuyst L. (2010). Community dynamics of bacteria in sourdough fermentations as revealed by their metatranscriptome. Appl. Environ. Microbiol..

[19] Weckx S., van der Meulen R., Maes D., Scheirlinck I., Huys G., Vandamme P., de Vuyst L. (2010). Lactic acid bacteria community dynamics and metabolite production of rye sourdough fermentations share characteristics of wheat and spelt sourdough fermentations. Food Microbiol..

[20] Corsetti A., Gobbetti M., Gänzle M.G. (2013). Technology of sourdough fermentation and sourdough applications. Handbook on Sourdough Biotechnology.

[21] Brandt M.J. (2014). Starter cultures for cereal based foods. Food Microbiol..

[22] Gaggiano M., Di Cagno R., de Angelis M., Arnault P., Tossut P., Fox P.F., Gobbetti M. (2007). Defined multi-species semi-liquid ready-to-use sourdough starter. Food Microbiol..

[23] Decock P., Cappelle S. (2005). Bread technology and sourdough technology. Trends Food Sci. Technol..

[24] Leroy F., de Vuyst L. (2004). Lactic acid bacteria as functional starter cultures for the food fermentation industry. Trends Food Sci. Technol..

[25] Comasio A., Harth H., Weckx S., de Vuyst L. (2019). The addition of citrate stimulates the production of acetoin and diacetyl by a citrate-positive *Lactobacillus crustorum* strain during wheat sourdough fermentation. Int. J. Food Microbiol..

[26] Perricone M., Bevilacqua A., Corbo M.R., Sinigaglia M. (2014). Technological characterization and probiotic traits of yeasts isolated from Altamura sourdough to select promising microorganisms as functional starter cultures for cereal-based products. Food Microbiol..

[27] Pontonio E., Nionelli L., Curiel J.A., Sadeghi A., Di Cagno R., Gobbetti M., Rizzello C.G. (2015). Iranian wheat flours from rural and industrial mills: Exploitation of the chemical and technology features, and selection of autochthonous sourdough starters for making breads. Food Microbiol..

[28] Ravyts F., de Vuyst L. (2011). Prevalence and impact of single-strain starter cultures of lactic acid bacteria on metabolite formation in sourdough. Food Microbiol..

[29] Rizzello C.G., Lorusso A., Montemurro M., Gobbetti M. (2016). Use of sourdough made with quinoa (*Chenopodium quinoa*) flour and autochthonous selected lactic acid bacteria for enhancing the nutritional, textural and sensory features of white bread. Food Microbiol..

[30] Ruiz Rodríguez L., Vera Pingitore E., Rollán G., Cocconcelli P.S., Fontana C., Saavedra L., Vignolo G., Hebert E.M. (2016). Biodiversity and technological-functional potential of lactic acid bacteria isolated from spontaneously fermented quinoa sourdoughs. J. Appl. Microbiol..

[31] Sterr Y., Weiss A., Schmidt H. (2009). Evaluation of lactic acid bacteria for sourdough fermentation of amaranth. Int. J. Food Microbiol..

[32] Vogelmann S.A., Seitter M., Singer U., Brandt M.J., Hertel C. (2009). Adaptability of lactic acid bacteria and yeasts to sourdoughs prepared from cereals, pseudocereals and cassava and use of competitive strains as starters. Int. J. Food Microbiol..

[33] Palla M., Agnolucci M., Calzone A., Giovannetti M., Di Cagno R., Gobbetti M., Rizzello C.G., Pontonio E. (2019). Exploitation of autochthonous Tuscan sourdough yeasts as potential starters. Int. J. Food Microbiol..

[34] Zheng J., Zhao X., Lin X.B., Gänzle M.G. (2015). Comparative genomics *Lactobacillus reuteri* from sourdough reveals adaptation of an intestinal symbiont to food fermentations. Sci. Rep..

[35] Camu N., de Winter T., Verbrugghe K., Cleenwerck I., Vandamme P., Takrama J.S., Vancanneyt M., de Vuyst L. (2007). Dynamics and biodiversity of populations of lactic acid bacteria and acetic acid bacteria involved in spontaneous heap fermentation of cocoa beans in Ghana. Appl. Environ. Microbiol..

[36] Hugas M., Garriga M., Aymerich T., Monfort J.M. (1993). Biochemical characterization of lactobacilli from dry fermented sausages. Int. J. Food Microbiol..

[37] Illeghems K., de Vuyst L., Weckx S. (2013). Complete genome sequence and comparative analysis of *Acetobacter pasteurianus* 386B, a strain well-adapted to the cocoa bean fermentation ecosystem. BMC Genomics.

[38] Laureys D., Aerts M., Vandamme P., de Vuyst L. (2019). The buffer capacity and calcium concentration of water influence the microbial species diversity, grain growth, and metabolite production during water kefir fermentation. Front. Microbiol..

[39] Laureys D., de Vuyst L. (2014). Microbial species diversity, community dynamics, and metabolite kinetics of water kefir fermentation. Appl. Environ. Microbiol..

[40] Moens F., Lefeber T., de Vuyst L. (2014). Oxidation of metabolites highlights the microbial interactions and role of *Acetobacter pasteurianus* during cocoa bean fermentation. Appl. Environ. Microbiol..

[41] Pelicaen R., Gonze D., Teusink B., de Vuyst L., Weckx S. (2020). Genome-scale metabolic reconstruction of *Acetobacter pasteurianus* 386B, a candidate functional starter culture for cocoa bean fermentation. Front. Microbiol..

[42] Ravyts F., Barbuti S., Frustoli M.A., Parolari G., Saccani G., de Vuyst L., Leroy F. (2008). Competitiveness and antibacterial potential of bacteriocin-producing starter cultures in different types of fermented sausages. J. Food Protect..

[43] Sánchez Mainar M., Weckx S., Leroy F. (2014). Coagulase-negative staphylococci favor conversion of arginine into ornithine despite a widespread genetic potential for nitric oxide synthase activity. Appl. Environ. Microbiol..

[44] Verce M., de Vuyst L., Weckx S. (2020). The metagenome-assembled genome of *Candidatus* Oenococcus aquikefiri from water kefir represents the species *Oenococcus sicerae*. Food Microbiol..

[45] Choi H., Kim Y.W., Hwang I., Kim J., Yoon S. (2012). Evaluation of *Leuconostoc citreum* HO12 and *Weissella koreensis* HO20 isolated from kimchi as a starter culture for whole wheat sourdough. Food Chem..

[46] De Vuyst L., Weckx S. (2016). The cocoa bean fermentation process: From ecosystem analysis to starter culture development. J. Appl. Microbiol..

[47] De Vuyst L., Leroy F. (2020). Functional role of yeasts, lactic acid bacteria, and acetic acid bacteria in cocoa fermentation processes. FEMS Microbiol. Rev..

[48] Papalexandratou Z., Lefeber T., Bahrim B., Lee O., Daniel H.M., de Vuyst L. (2013). *Hanseniaspora opuntiae*, *Saccharomyces cerevisiae*, *Lactobacillus fermentum*, and *Acetobacter pasteurianus* predominate during well-performed Malaysian cocoa bean box fermentations, underlining the importance of these microbial species for a successful cocoa bean fermentation process. Food Microbiol..

[49] Ravyts F., de Vuyst L., Leroy F. (2012). Bacterial diversity and functionalities in food fermentations. Eng. Life Sci..

[50] Laureys D., Aerts M., Vandamme P., de Vuyst L. (2018). Oxygen and diverse nutrients influence the water kefir fermentation process. Food Microbiol..

[51] Laureys D., de Vuyst L. (2017). The water kefir grain inoculum determines the characteristics of the resulting water kefir fermentation process. J. Appl. Microbiol..

[52] Li Z., Li H., Bian K. (2016). Microbiological characterization of traditional dough fermentation starter (jiaozi) for steamed bread making by culture-dependent and culture-independent methods. Int. J. Food Microbiol..

[53] Minervini F., Lattanzi A., de Angelis M., Di Cagno R., Gobbetti M. (2012). Influence of artisan bakery- or laboratory-propagated sourdoughs on the diversity of lactic acid bacterium and yeast microbiotas. Appl. Environ. Microbiol..

[54] Zhang G., He G. (2013). Predominant bacteria diversity in Chinese traditional sourdough. J. Food Sci..

[55] Hermann M., Petermeier H., Vogel R.F. (2015). Development of novel sourdoughs with in situ formed exopolysaccharides from acetic acid bacteria. Eur. Food Res. Technol..

[56] Ua-Arak T., Jakob F., Vogel R.F. (2016). Characterization of growth and exopolysaccharide production of selected acetic acid bacteria in buckwheat sourdoughs. Int. J. Food Microbiol..

[57] Ua-Arak T., Jakob F., Vogel R.F. (2017). Influence of levan-producing acetic acid bacteria on buckwheat-sourdough breads. Food Microbiol..

[58] Ripari V., Cecchi T., Berardi E. (2016). Microbiological characterisation and volatiles profile of model, ex-novo, and traditional Italian white wheat sourdoughs. Food Chem..

[59] Ravyts F., Vrancken G., D’Hondt K., Vasilopoulos C., de Vuyst L., Leroy F. (2009). Kinetics of growth and 3-methyl-1-butanol production by meat-borne, coagulase-negative staphylococci in view of sausage fermentation. Int. J. Food Microbiol..

[60] Ravyts F., Steen L., Goemaere O., Paelinck H., de Vuyst L., Leroy F. (2010). The application of staphylococci with flavour-generating potential is affected by acidification in fermented dry sausages. Food Microbiol..

[61] Sánchez Mainar M., Stavropoulou D.A., Leroy F. (2017). Exploring the metabolic heterogeneity of coagulase-negative staphylococci to improve the quality and safety of fermented meats: A review. Int. J. Food Microbiol..

[62] Stavropoulou D.A., Borremans W., de Vuyst L., de Smet S., Leroy F. (2015). Amino acid conversions by coagulase-negative staphylococci in a rich medium: Assessment of inter- and intraspecies heterogeneity. Int. J. Food Microbiol..

[63] Verce M., de Vuyst L., Weckx S. (2020). Comparative genomics of *Lactobacillus fermentum* suggests a free-living lifestyle of this lactic acid bacterial species. Food Microbiol..

[64] Meroth C.B., Walter J., Hertel C., Brandt M.J., Hammes W.P. (2003). Monitoring the bacterial population dynamics in sourdough fermentation processes by using PCR-denaturing gradient gel electrophoresis. Appl. Environ. Microbiol..

[65] Papalexandratou Z., Falony G., Romanens E., Jimenez J.C., Amores F., Daniel H.M., de Vuyst L. (2011). Species diversity, community dynamics, and metabolite kinetics of the microbiota associated with traditional Ecuadorian spontaneous cocoa bean fermentations. Appl. Environ. Microbiol..

[66] Spitaels F., Wieme A.D., Janssens M., Aerts M., Daniel H.-M., VAN Landschoot A., de Vuyst L., Vandamme P. (2014). The microbial diversity of traditional spontaneously fermented lambic beer. PLoS ONE.

[67] Ercolini D., Pontonio E., de Filippis F., Minervini F., La Storia A., Gobbetti M., Di Cagno R. (2013). Microbial ecology dynamics during rye and wheat sourdough preparation. Appl. Environ. Microbiol..

[68] Van Kerrebroeck S., Comasio A., Harth H., de Vuyst L. (2018). Impact of starter culture, ingredients, and flour type on sourdough bread volatiles as monitored by selected ion flow tube-mass spectrometry. Food Res. Int..

[69] Van Kerrebroeck S., Harth H., Comasio A., de Vuyst L. (2018). Monitoring of starter culture-initiated liquid wheat and teff sourdough fermentations by selected ion flow tube-mass spectrometry. J. Sci. Food Agric..

[70] Lefeber T., Papalexandratou Z., Gobert W., Camu N., de Vuyst L. (2012). On-farm implementation of a starter culture for improved cocoa bean fermentation and its influence on the flavour of chocolates produced thereof. Food Microbiol..

[71] Illeghems K., de Vuyst L., Weckx S. (2015). Comparative genome analysis of the candidate functional starter culture strains *Lactobacillus fermentum* 222 and *Lactobacillus plantarum* 80 for controlled cocoa bean fermentation processes. BMC Genomics.

[72] Vrancken G., Rimaux T., de Vuyst L., Leroy F. (2008). Kinetic analysis of growth and sugar consumption by *Lactobacillus fermentum* IMDO 130101 reveals adaptation to the acidic sourdough ecosystem. Int. J. Food Microbiol..

[73] Rimaux T., Vrancken G., Pothakos V., Maes D., de Vuyst L., Leroy F. (2011). The kinetics of the arginine deiminase pathway in the meat starter culture *Lactobacillus sakei* CTC 494 are pH-dependent. Food Microbiol..

[74] Rimaux T., Vrancken G., Vuylsteke B., de Vuyst L., Leroy F. (2011). The pentose moiety of adenosine and inosine is an important energy source for the fermented-meat starter culture *Lactobacillus sakei* CTC 494. Appl. Environ. Microbiol..

[75] Cousin F.J., Le Guellec R., Chagnot C., Goux D., Dalmasso M., Laplace J.M., Cretenet M. (2019). *Oenococcus sicerae* sp. nov., isolated from French cider. Syst. Appl. Microbiol..

[76] Laureys D., Cnockaert M., de Vuyst L., Vandamme P. (2016). *Bifidobacterium aquikefiri* sp. nov., isolated from water kefir. Int. J. Syst. Evol. Microbiol..

[77] Verce M., de Vuyst L., Weckx S. (2019). Shotgun metagenomics of a water kefir fermentation ecosystem reveals a novel *Oenococcus* species. Front. Microbiol..

[78] Zheng J., Ruan L., Sun M., Gänzle M.G. (2015). A genomic view of lactobacilli and pediococci demonstrates that phylogeny matches ecology and physiology. Appl. Environ. Microbiol..

[79] Edwards C.G., Collins M.D., Lawson P.A., Rodriguez A.V. (2000). *Lactobacillus nagelii* sp.nov., an organism isolated from a partially fermented wine. Int. J. Syst. Evol. Microbiol..

[80] Stavropoulou D.A., de Vuyst L., Leroy F. (2018). Nonconventional starter cultures of coagoulase-negative staphylococci to produce animal-derived fermented foods, a SWOT analysis. J. App. Microbiol..

[81] Matsushita K., Toyama H., Tonouchi N., Okamoto-Kainuma A. (2016). Acetic Acid Bacteria: Ecology and Physiology.

[82] Prust C., Hoffmeister M., Liesegang H., Wiezer A., Fricke W.F., Ehrenreich A., Gottschalk G., Deppenmeier U. (2005). Complete genome sequence of the acetic acid bacterium *Gluconobacter oxydans*. Nat. Biotechnol..

[83] Gupta A., Singh V.K., Qazi G.N., Kumar A. (2001). *Gluconobacter oxydans*: Its biotechnological applications. J. Mol. Microbiol. Biotechnol..

[84] Claret C., Salmon J.M., Romieu C., Bories A. (1994). Physiology of *Gluconobacter oxydans* during dihydroxyacetone production from glycerol. Appl. Microbiol. Biotechnol..

[85] Hu Z.C., Zheng Y.G., Schen Y.C. (2010). Dissolved-oxygen-stat fed-batch fermentation of 1,3-dihydroxyacetone from glycerol by *Gluconobacter oxydans* ZJB09112. Biotechnol. Bioprocess Eng..

